# Programmed Death Ligand 2 in Cancer-Induced Immune Suppression

**DOI:** 10.1155/2012/656340

**Published:** 2012-04-29

**Authors:** Esdy N. Rozali, Stanleyson V. Hato, Bruce W. Robinson, Richard A. Lake, W. Joost Lesterhuis

**Affiliations:** ^1^Tumor Immunology Group, School of Medicine and Pharmacology, University of Western Australia, 4th Floor, G-block, Sir Charles Gairdner Hospital, Hospital Avenue, Nedlands, WA 6009, Australia; ^2^Department of Tumor Immunology, Nijmegen Centre for Molecular Life Sciences, Radboud University Nijmegen Medical Centre, P.O. Box 9101, 6500 HB Nijmegen, The Netherlands; ^3^National Centre for Asbestos Related Diseases, The University of Western Australia, 35 Stirling Highway, Crawley, WA 6009, Australia; ^4^Department of Medical Oncology, Radboud University Nijmegen Medical Centre, P.O. Box 9101, 6500 HB Nijmegen, The Netherlands

## Abstract

Inhibitory molecules of the B7/CD28 family play a key role in the induction of immune tolerance in the tumor microenvironment. The programmed death-1 receptor (PD-1), with its ligands PD-L1 and PD-L2, constitutes an important member of these inhibitory pathways. The relevance of the PD-1/PD-L1 pathway in cancer has been extensively studied and therapeutic approaches targeting PD-1 and PD-L1 have been developed and are undergoing human clinical testing. However, PD-L2 has not received as much attention and its role in modulating tumor immunity is less clear. Here, we review the literature on the immunobiology of PD-L2, particularly on its possible roles in cancer-induced immune suppression and we discuss the results of recent studies targeting PD-L2 in cancer.

## 1. Introduction

Molecules of the B7-CD28 family play an important role in T-cell activation and tolerance. These pathways are not only responsible for providing positive costimulatory signals to sustain T-cell activity, but also contribute inhibitory signals that modulate the magnitude of T-cell responses [1]. Useful as this negative feedback may be during physiological homeostasis, it may be a problem in the context of cancer. It is now clear that the inhibitory members of the B7-CD28 family are upregulated by a variety of cells within the tumor microenvironment [2]. Thus, the selective blockade of these inhibitory molecules is an attractive approach to cancer immunotherapy.

The programmed death-1 receptor (PD-1, CD279) with its ligands PD-L1 (CD274, B7-H1) and PD-L2 (CD273, B7-DC) constitutes one such inhibitory pathway. Therapeutic antibodies for blocking PD-1 and PD-L1 have been developed and are undergoing human clinical testing [3, 4]. Negating the PD-1/PD-L1 interaction is of particular interest as PD-L1 is upregulated by many human cancers [5]. On the other hand, the role of PD-L2 in modulating immune responses is less clear, and its expression is more restricted compared to PD-L1, thus making it a less obvious target in cancer immunotherapy. However, in this context, several aspects of PD-L2 biology deserve attention, including a partial contextual dependency of PD-L2 expression. Recent reviews have discussed the importance of PD-L1 in tumor immunology [4, 6]. Here, we will focus on the immunobiology of PD-L2 and particularly on its possible roles in cancer-induced immune suppression.

## 2. Expression Pattern of PD-L2

The patterns of expression of PD-L1 and PD-L2 are quite distinct. PD-L1 is constitutively expressed by a wide variety of immune cells and nonimmune cells and most normal tissue cells seem to be able to upregulate PD-L1 in the presence of strong inflammatory signals, presumably to prevent collateral damage induced by potent but potentially destructive Th1/17 T-cell responses [7–10]. Compared to PD-L1, constitutive basal expression of PD-L2 is low. PD-L2 expression was initially thought to be restricted to antigen-presenting cells such as macrophages and dendritic cells (DCs) [11]. In recent years however, several groups have shown that PD-L2 expression can be induced on a wide variety of other immune cells and nonimmune cells depending on microenvironmental stimuli [12–17].

**Figure 1:**
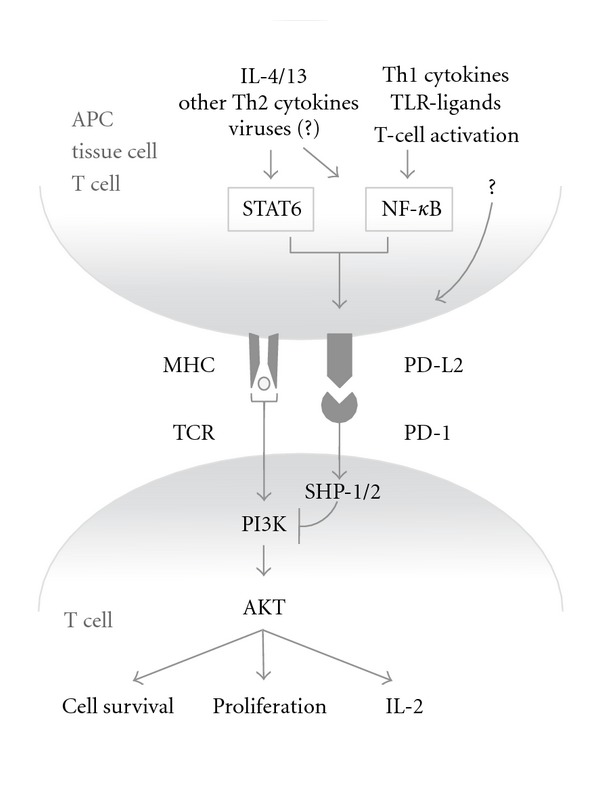
PD-L2/PD-1 signaling. PD-L2 expression by different cell types is regulated by STAT6 and NF-*κ*B, although other possible regulators cannot be excluded. The most potent inducers of PD-L2 expression appear to be Th2 cytokines, particularly IL-4. Several new activators of STAT6 (such as viruses) have been found, but whether they therefore also upregulate PD-L2 is not known yet. PD-L2/PD-1 interaction results in the suppression of TCR-induced PI3K/AKT activation and subsequent attenuation of T cell survival, cytokine production and proliferation.

Exposure of DCs and macrophages to Th2 (IL-4) cytokines increased the expression of PD-L2 as did IFN*γ* and toll-like receptor ligands [17, 18] (Figure 1). In addition, cytokines binding to receptors that use the common *γ*-chain such as IL-2, IL-7, IL-15, and IL-21 upregulated PD-L2 in these cells [12]. Alveolar epithelial cells express high levels of PD-L2 in the presence of IL-4 when infected with respiratory syncytial virus [10]. Constitutive expression of PD-L2 on human umbilical vein endothelial cells has been observed and stimulation by IFN*γ* and TNF*α* *in vitro* further enhanced its expression [19]. Also human colonic fibroblasts have been shown to express PD-L2, resulting in T-cell suppression in the gut epithelial mucosa [9]. Of special importance to the field of tumor immunology is the finding that not only normal fibroblasts, but also cancer-associated fibroblasts can constitutively express PD-L2 (further discussed below) [20]. Recently, constitutive expression of PD-L2 was found on 50–70% of mouse peritoneal CD5^+^ B cells and PD-L2 expression was found to be unique to this particular subset of B cells [13]. An additional level of complexity was discovered in the finding that T cells themselves can upregulate PD-L2 upon activation *in vitro* [21, 22]. We have shown that this was predominantly the case for Th2 cells activated in the presence of IL-4, and less so for Treg, Th1, and Th17 cells [23]. From these data a new picture is emerging in which the expression of PD-L2 is much less restricted than previously thought and at least for some cells partly depends on microenvironmental cues, with a specific role for Th2 cytokines.

## 3. Regulation of PD-L2 Expression

From the data discussed above, it can be inferred that signalling pathways downstream of cytokine receptors and innate immune activators play an important role in the regulation of PD-L2 expression. Indeed, two major pathways that have been reported to regulate PD-L2 expression are the NF*κ*B-pathway and the signal transducer and activator of transcription (STAT) 6 pathway (Figure 1). Two groups have found that macrophages from Stat6^−/−^ mice are unable to express PD-L2 [24, 25]. These results were confirmed in bone-marrow-derived DCs from Stat6^−/−^ mice and in human monocyte-derived DCs in which STAT6 was pharmacologically inhibited [17]. STAT6 is a signaling molecule and transcription factor that is especially important in the regulation of Th2 immune responses and it is activated by ligation of the IL-4 and IL-13 receptor with its ligands IL-4 or IL-13 [26]. Recently, also the cytokines TSLP and IL-27 have been shown to activate STAT6, as well as viruses in a JAK-independent manner, providing the possibility that these stimuli may also induce PD-L2 expression [27–29].

NF*κ*B was shown to play a role in the regulation of PD-L2 expression by Liang and colleagues: although knockdown of NF*κ*B did not completely abrogate PD-L2 expression, DCs from NF-*κ*B p50^−/−^ p65^−/+^ mice had lower levels of expression and were less able to upregulate PD-L2 when stimulated with exogenous IFN*γ* or LPS [30]. However, NF-*κ*B p50^−/−^ mice are severely hampered in the production of the STAT6 activating cytokines IL-4 and IL-13 [31], possibly explaining the lowered PD-L2 expression found by Liang and colleagues. Indeed, in contrast to the findings by Liang et al., another study found that the PD-L2 promoter could be activated by IL-4 signaling but not by LPS signaling, a strong NF-*κ*B inducer [32]. Thus, it seems that NF-*κ*B does not play a direct role in the induction of PD-L2 expression. However, an indirect role cannot be ruled out since at least one study showed that pharmacological blocking of NF-*κ*B interfered with STAT6 DNA binding but not phosphorylation or nuclear translocation, indicating that NF-*κ*B might have a role in regulating STAT6 DNA binding activity and thus indirectly controls PD-L2 expression [33]. Together, these findings hint at the possibility that PD-L2 may be of particular importance in the regulation of Th2 type immune responses. Whether the NF*κ*B and STAT6 pathways are the only pathways that are of importance for the regulation of PD-L2 expression remains an open question.

## 4. Molecular Consequences of PD-L2/PD-1 Interactions

The structures of PD-1/PD-L1 [34] and PD-1/PD-L2 [35] reveal differences in the binding modalities, which helps explain the distinct molecular mechanisms of interaction between PD-1 and its ligands. By investigating PD-1 interactions with its ligands by surface plasmon resonance and cell surface binding, Ghiotto et al. showed that while PD-L2 interact in a direct manner with PD-1, PD-L1 binding to PD-1 involves complex conformational changes. The notion of PD-L1 and PD-L2 simultaneously binding to PD-1 was also dispelled, indicating that the two ligands cross-compete to bind to the receptor [36]. Furthermore, the relative affinity of PD-L2 to PD-1 was calculated to be 2–6-fold higher than that of PD-L1 [37]. If expressed at the same level, PD-L2 would be expected to outcompete PD-L1 for binding PD-1, but the physiological relevance of this competition is not yet fully clear [38]. The fact that PD-L2 is generally expressed at a lower level may favour PD-L1 as the primary binding ligand of PD-1, except for during Th2 responses when PD-L2 is upregulated.

PD-1/PD-L interactions lead to phosphorylation of two tyrosines at the intracellular tail of PD-1. These tyrosines are part of an Immunoreceptor Tyrosine-based Inhibitory Motif (ITIM) and an Immunoreceptor Tyrosine-based Switch Motif (ITSM). ITSM then recruits either of two structurally related protein tyrosine phosphatases, SH2-domain containing tyrosine phosphatase 1 (SHP-1) and SHP-2 [39], which suppress activation of PI3K/Akt [40]. Consequently, the survival factor Bcl-xL is downregulated [40] and expression of transcription factors associated with effector cell function including GATA-3, T-bet and Eomes are lost [41]. The net result of these PD-1-induced cascades is an impairment of proliferation, cytokine production, cytolytic function, and survival of the T cell [42].

Whether PD-L2 can induce signalling downstream of its intracellular domain has not been well characterized. Using magnetic beads coated with anti-CD3 and anti-CD28 as artificial antigen-presenting cells, Messal and colleagues found that when PD-L2 on T cells was ligated with the same beads coated with anti-PD-L2, T-cell proliferation and production of IL-2, IL-10, and IFN*γ* was significantly decreased [21]. Studies from a different group demonstrated that cross-linking of PD-L2 on T cells that were transduced with PD-L2 siRNA resulted in the elimination of the negative effect on IFN*γ* production [22]. These data indicate that indeed PD-L2 does induce signalling downstream and as such plays a role in the modulation of T-cell function, but the exact molecular pathway is yet to be elucidated.

Of note, PD-1 may not be the only receptor for PD-L2. This can be inferred from helminth infection and allergic animal models, showing enhanced disease severity when PD-L2 blocking antibodies were used, but not when PD-1 blocking antibodies were used [43, 44]. Furthermore, PD-L2 mutants with abolished PD-1 binding capacity could still exert functional effects on T cells from normal and PD-1-deficient mice [45]. Thus, although for PD-L1 another receptor has been found in B7.1, for PD-L2 this still remains enigmatic [46].

## 5. Physiological Function of PD-L2

The initial finding of enhanced expression of PD-L2 on activated professional antigen-presenting cells suggested that PD-L2 mainly functioned in the induction phase of T-cell immunity, whereas PD-L1 which is much more widely expressed, played an important role in constraining activated T cells at the effector site. However, the above-mentioned data showing a wider inducible expression of PD-L2 as well as *in vivo* animal studies have demonstrated that PD-L2 probably functions both at the induction phase as well as the effector phase of T-cell responses. For example, antigen-presenting cells from PD-L2^−/−^ mice displayed an enhanced T-cell activating potential both *in vitro* and *in vivo* [47]. Inducible experimental autoimmune encephalitis models have shown that therapeutic blockade of PD-L2 results in enhanced disease severity not only when the antibodies were administered at the time of disease initiation, but also in the chronic phase [48, 49].

The physiological role of dampening and regulating T-cell responsiveness seems especially important in the mucosal immune response against environmental antigens [50]. For example, in PD-L2^−/−^ mice experimentally induced oral tolerance to chicken ovalbumin was abrogated, and animal models using exposure to environmental allergens through the lung mucosa demonstrate enhanced airway hypersensitivity when PD-L2 (but not PD-L1) is knocked out or pharmacologically blocked [43, 47, 51]. Experimental colitis models, however, have thus far not demonstrated a role for PD-L2 in controlling disease severity [52]. A possible explanation for this lack of effect could be that the colitis induction in these models (adoptive transfer of CD4^+^CD45RB^high^ T cells into SCID mice) probably does not involve a Th2-skewed microenvironment. Although immune infiltrates in human ulcerative colitis have been shown to highly express PD-L1 [52], this has not been investigated for PD-L2.

As can be inferred from the above-mentioned asthma models, as well as from helmintic infection animal models demonstrating enhanced disease severity in the absence of PD-L2 signalling [44], PD-L2 appears to predominantly be of significance in the modulation of Th2 immune responses. Animal models of Th1-driven diseases, without a dominant Th2 component, such as autoimmune diabetes have generally shown a more dominant role of PD-L1 over PD-L2 in restraining T-cell activity and prevention of subsequent collateral tissue damage [53, 54].

Intriguingly, in a PD-L2 knock out mouse model different from the previously discussed ones [47, 51], IFN*γ*-production by T-helper cells as well as IFN*γ*-dependent humoral responses and antigen-specific CTL responses were impaired, indicating that PD-L2 also functions as a tuning molecule that can even augment CTL and Th1 responses [55]. However, using an *in vitro* system of engineered T-cell stimulator cells that detached PD-L2/PD-1 interactions from the context of other molecules regulating T-cell activation, no positive costimulatory role for PD-L2 was found [56].

Although the final verdict is still out, and PD-L1 and 2 do appear to have overlapping effects, together these data indicate that the main physiological function of PD-L2 could lie in the dampening and regulation of Th2-driven T-cell immune responses both during the induction and the effector phase, with possibly special significance in mucosal responses against environmental antigens. However, given the fact that PD-L2 also inhibits IFN*γ* production by Th1 cells, and Th2 responses appear to prevent acute tissue damage by Th1 or Th17 cells, as has been shown very recently in a helminth model [57], it could be hypothesized that although the Th2 response is the “driver” of PD-L2 expression, the potentially destructive Th1/17 component of the local immune response is the eventual target.

## 6. PD-L2 in Cancer

Since PD-L2 appears to play an important role in the modulation of Th2 responses, while in the context of antitumor immunity Th1 responses are the most potent, it does not seem obvious to choose PD-L2 as a target in cancer. However, in recent years evidence has accumulated showing that tumor microenvironments are often deviated towards an ineffective Th2 type of immune milieu, resulting in cancer cell escape from immune surveillance. For example, breast cancer cells have been shown to produce IL-13 themselves, resulting not only in autocrine STAT6-phosphorylation but also in the instruction of DCs to skew CD4 T cells towards a Th2 phenotype with high production of IL-4 and IL-13 [58]. In addition, human and murine studies in pancreatic cancer have shown high local production of TSLP (another STAT6-activating cytokine [27]), resulting in Th2 skewing and enhanced tumor outgrowth [59, 60]. PD-L2 upregulation in response to local Th2 cytokines may thus affect tumor-specific CTL reactivity, either in the induction phase in the tumor-draining lymph node or in the effector phase in the tumor. Hence, there is a clear rationale to further investigate the relevance of PD-L2 in cancer.

### 6.1. Clinical Relevance of PD-L2 Expression in Cancer

Given the possible immune evasion to antigen-specific T cells by PD-L2-expressing tumor cells, several groups have investigated the possible correlation between tumor PD-L expression and clinical outcome in retrospective patient cohorts. These studies were performed before the observation was made that also cancer-associated fibroblasts upregulate both PD-L1 and 2 [20], and therefore a clear distinction between tumor cell and tumor stroma expression may not have been made. Ohigashi et al. [61] investigated the expression of PD-L1 and PD-L2 in human esophageal cancer to determine their clinical significance in patients prognosis after surgery. Using RT-qPCR and immunohistochemistry, the authors showed that both PD-L1 and PD-L2 are expressed in frozen tissue samples of esophageal cancer patients and PD-L2-positive patients had a poorer prognosis than the negative patients, as was the case for PD-L1 [61]. Interestingly, there was a significant inverse correlation between PD-L2 expression and CD8 TILs but not CD4 TILs. In a retrospective study involving 51 patients with pancreatic cancer, 27% of the analyzed tumors expressed PD-L2 versus 39% expressing PD-L1. No correlation was found between PD-L2 expression and survival, whereas PD-L1 expression correlated with an impaired survival [62]. Similarly, in a cohort of 70 patients with ovarian cancer, the majority of the tumors were negative or weakly positive and although PD-L2 expression was correlated with an impaired survival, this did not reach statistical significance [63]. And lastly, in a study involving 125 patients with hepatocellular carcinoma a minority had high PD-L2 expression, and again, although PD-L2 expression was correlated with an impaired disease-free survival, this difference was not statistically significant [64].

Thus, the majority of studies have found a significant correlation between impaired survival and PD-L1 expression, but much less so for PD-L2. Although several studies have found an impaired survival in patients with PD-L2 expressing tumors, this reached statistical difference in only one of these studies [61]. However, it is important to note that in the majority of studies PD-L2 was expressed in only a minority of patients. In addition, it is not inconceivable that PD-L2 expression is more dependent on environmental cues than PD-L1, which seems to be expressed in a more constitutive manner, although this can be further upregulated with proinflammatory stimuli [65]. In fact, if the PD-L's are induced in response to IFN*γ* that is produced by antigen-specific tumor-infiltrating T cells, a process recently termed adaptive resistance [4], this may actually reflect a positive event in the context of antitumor immunity, but this does make the data more difficult to interpret. Finally, there are some technical issues with different findings depending on whether frozen sections or paraffin-embedded slides were used, with a higher percentage of positive tumors from frozen sections, as has been shown for PD-L1 [66]. Thus for these reasons, although PD-L1 may indeed be the more dominant negative inhibitory molecule in the context of tumor immunology, PD-L2 should not yet be dismissed as a possible second important suppressive molecule in the tumor microenvironment.

It is also important to note here that perhaps not only PD-L2 expression by the tumor cells themselves, but rather by stromal cells is of importance. Nazareth and colleagues found constitutively high PD-L1 and 2 expression in fibroblasts that were cultured from human non-small-cell lung cancers [20]. This expression appeared to be functional, since *in vitro* blocking studies demonstrated that the fibroblasts inhibited IFN*γ*-production by autologous T cells in a PD-L1- and 2-dependent manner. For this reason, future studies should not only focus on PD-L expression by tumor cells only, but also by the tumor stroma.

### 6.2. Therapeutic Studies Targeting PD-L2 in Cancer

Given its potential role in cancer-associated immune suppression in the tumor microenvironment, targeting the PD-1/PD-L pathway seems an attractive treatment strategy. Several studies have investigated the therapeutic effect of blocking antibodies against the PD-1/PD-L pathway in murine cancer models, demonstrating enhanced tumor control rates, though in none of these studies the blocking of PD-L2 was used as a defined treatment strategy [67–70]. Although in a few studies PD-L2 blocking strategies were used, this was always in combination with the targeting of PD-L1 [71, 72]. In these studies again impaired tumor outgrowth was demonstrated. The true additive value of adding anti-PD-L2 on top of anti-PD-L1 cannot be assessed based on these studies, since separate single-antibody treatments were not tested.

In one study using the Panc02 murine pancreatic tumor model, decreased tumor outgrowth rates on day 21 were seen when the animals were treated with PD-L2 blocking antibodies, comparable to that seen with blocking PD-L1 or PD-1 alone [73]. In contrast with these data, in a hepatic metastasis model of CT-26 colon cancer, PD-L2^−/−^ mice displayed impaired survival and increased tumor outgrowth in combination with a decreased tumor-specific CTL response [55]. It is difficult to reconcile these conflicting data, but the difference in outcome may be the result of a difference in mouse strain backgrounds or differences in the local tumor microenvironment and cytokine milieu that influence PD-L2 expression by its several constituent cell types.

Human data about targeting PD-L2 in cancer are scarce. Currently a phase I study is ongoing investigating AMP-224, a recombinant fusion protein of PD-L2 and the Fc portion of IgG1 (http://ClinicalTrials.gov Identifier NCT01352884) [74]. Although there are no results to date about specifically targeting PD-L2 in humans, promising results have been seen with antibodies targeting PD-1 with objective responses in several types of cancer and with tolerable toxicity, specifically autoimmune-related adverse events [3, 4]. In addition, several groups have used approaches other then antibodies to target PD-L2 in humans. Hobo and colleagues used siRNA to knock down PD-L1 and PD-L2 in DCs, with the ultimate goal of incorporating this approach in DC-based cancer vaccines. PD-L2-silenced DCs modestly improved IFN*γ* production by allogeneic T cells, but double knockdown of both PD-Ls resulted in a synergistic increase of IFN*γ* production and proliferation capacity of antigen-specific T cells *in vitro* [75]. This was also followed by a synergistic improvement of cytokine production in double PD-L blockade compared to single PD-L1 knockdown or PD-L2 knockdown [75].

Recently, we found that platinum-based chemotherapeutic drugs that form the cornerstone in the medical treatment of many cancers, dephosphorylate STAT6, resulting in downregulation of PD-L2 by DCs [17]. We found that this resulted in an enhanced T-cell activating potential of the DCs in vitro. Moreover, also tumor cells downregulated PD-L2 when treated with platinum drugs, resulting in enhanced CTL recognition. Indeed tumor STAT6 expression correlated strongly with an enhanced recurrence-free survival in a cohort of patients with head and neck cancer that had been treated with cisplatin-based chemoradiation. Conversely, in a cohort of patients that had been treated with radiotherapy alone, STAT6 expression showed a clear trend towards a poor clinical outcome, which could be explained by the immune-evasive potential of STAT6-expressing tumor cells, if not attacked by platinum. Although in this study it could not be ruled out that STAT6-dependent effects other than PD-L2 upregulation also played a role, these results indicate that we may in fact already be targeting PD-L2 in cancer patients with one of the clinically most widely used groups of chemotherapeutics [76].

However, to truly determine whether PD-L2 is a relevant molecule to target in cancer immunotherapy, more studies are necessary. Given the dependency of PD-L2 expression on environmental cues, the outcomes may differ between tumor models and tumor types in animals and humans, or even between patients with the same tumor type. Future studies should investigate whether it is possible to predict which patients might respond to PD-L2 blockage by first defining the type of immune response occurring in the tissue, for example, whether it is Th2 or not. In addition, double blockade combining PD-L1 and 2, or combining anti-PD-L2 with anti-CTLA4, which blocks an immune checkpoint more during the induction phase could potentially be more efficient [67]. Finally, since several forms of cancer chemotherapy have been shown not only to induce antigen release but also subsequent immune activation [77, 78], the therapeutic efficacy of these drugs could potentially be further enhanced by combining it with PD-L2 blockade.

## 7. Conclusion

It has now been demonstrated that PD-L2 is principally an inhibitory molecule, expressed not only by antigen-presenting cells, but also by other immune cells including T cells themselves and nonimmune cells in an inducible manner, mainly through Th2-associated cytokines. Based on the current literature, it is not yet possible to draw a definite conclusion on the relevance of PD-L2 in the immune-suppressive tumor microenvironment, although there are some encouraging data indicating that targeting PD-L2 in cancer may be a viable treatment approach. Therefore, more studies targeting PD-L2 in the context of antitumor immunity are urgently needed.
